# Supplementary material for: Acute mental health responses during the COVID-19 pandemic in Australia
Source: PLoS One. 2020 Jul 28;15(7):e0236562. doi: 10.1371/journal.pone.0236562 (PMC7386645; doi:10.1371/journal.pone.0236562)
Supplement: S1 File — (DOCX) [file pone.0236562.s001.docx]

**S1 File. Advertisement used for recruitment on Facebook, LinkedIn and Twitter.**

Facebook and LinkedIn:

In order to understand more about the impacts of Coronavirus on the mental health of all Australians, we're inviting you to take part in an online study. The study will involve completing 3 questionnaires (15 mins each) - one now, in 2 weeks, and in 4 weeks’ time. To say thank you, participants will go into the draw to win 1 of 5 Prezzee gift cards.

Twitter

Researchers at @UNSW and @blackdoginst are interested in understanding the impact of COVID-19 on the #mentalhealth of Australians. Complete 3 x 15 minute surveys and go in the draw to win. Get started here: blackdoginstitute.org.au/coronavirus-survey #coronavirusau #Covid_19australia
