# Supplementary material for: Acute mental health responses during the COVID-19 pandemic in Australia
Source: PLoS One. 2020 Jul 28;15(7):e0236562. doi: 10.1371/journal.pone.0236562 (PMC7386645; doi:10.1371/journal.pone.0236562)
Supplement: S1 Table — (DOCX) [file pone.0236562.s002.docx]

**S1 Table. Mental health in people with and without a prior self-reported mental health diagnosis.**

|  | **Prior mental health diagnosis** | | | **No prior mental health diagnosis** | | |  |
| --- | --- | --- | --- | --- | --- | --- | --- |
|  | **N** | **Mean** | **SD** | **N** | **Mean** | **SD** | **Independent samples *t* test** |
| **Uncertain about future** | 3581 | 3.57 | 1.07 | 1351 | 3.21 | 1.05 | t (4930) = 10.63, p = 0.00 |
| **Lonely** | 3581 | 2.83 | 1.29 | 1351 | 2.23 | 1.16 | t (4930) = 14.89, p = 0.00 |
| **Worry about finances** | 3581 | 2.83 | 1.26 | 1351 | 2.41 | 1.19 | t (4930) = 10.68, p = 0.00 |
| **Worry about contracting COVID-19** | 3574 | 2.89 | 1.08 | 1344 | 2.71 | 1.03 | t (4916) = 5.23, p = 0.00 |
| **Perceived likelihood** | 3575 | 49.04 | 24.88 | 1347 | 45.97 | 24.61 | t (4920) = 3.87, p = 0.00 |
| **Perceived control** | 3574 | 71.05 | 19.79 | 1346 | 73.41 | 19.25 | t (4918) = -3.76, p = 0.00 |
| **Severity of illness** | 3564 | 3.44 | 1.07 | 1341 | 3.16 | 1.02 | t (4903) = 8.39, p = 0.00 |
| **Worry about loved ones contracting COVID-19** | 3581 | 3.59 | 1.03 | 1351 | 3.38 | 1.02 | t (4930) = 6.22, p = 0.00 |
| **Self-rated health** | 3481 | 2.85 | 0.94 | 1310 | 3.39 | 9.40 | t (4789) = 17.73, p = 0.00 |
| **DASS-21 Total** | 3567 | 45.52 | 25.26 | 1345 | 26.57 | 18.93 | t (4910) = 25.00, p = 0.00 |
| **DASS-21 Depression** | 3567 | 16.22 | 10.85 | 1345 | 8.87 | 7.70 | t (4910) = 22.78, p = 0.00 |
| **DASS-21 Anxiety** | 3567 | 10.47 | 8.50 | 1345 | 5.12 | 5.98 | t (4910) = 21.19, p = 0.00 |
| **DASS-21 Stress** | 3567 | 18.83 | 9.44 | 1345 | 12.58 | 8.12 | t (4910) = 21.49, p = 0.00 |
| **Whiteley-6 (health anxiety)** | 3575 | 13.93 | 5.75 | 1351 | 11.19 | 4.74 | t (4924) = 15.63, p = 0.00 |
| **Contamination Fears** | 3483 | 11.42 | 9.05 | 1319 | 9.12 | 7.87 | t (4800) = 8.14, p = 0.00 |
| **AUDIT-C Total (alcohol)** | 3411 | 3.10 | 2.72 | 1289 | 3.23 | 2.44 | t (4698) = -1.45, p = 0.15 |
| **PAVS Total (physical activity)** | 3429 | 170.90 | 360.41 | 1289 | 226.32 | 393.88 | t (4716) = -4.59, p = 0.00 |
|  |  | **n** | **%** |  | **n** | **%** |  |
| **Whiteley-6 (health anxiety)** |  | 923 | 25.8 |  | 146 | 10.8 | ꭓ^2^ (1) = 130.03 p <.001 |
| **AUDIT-C (hazardous drinking)** |  | 1742 | 48.6 |  | 737 | 54.6 | ꭓ^2^ (1) = 52.52 p <.001 |
| **PAVS (inactive)** |  | 1349 | 58.1 |  | 631 | 49.0 | ꭓ^2^ (1) = 13.99 p <.001 |
