# Supplementary material for: Acute mental health responses during the COVID-19 pandemic in Australia
Source: PLoS One. 2020 Jul 28;15(7):e0236562. doi: 10.1371/journal.pone.0236562 (PMC7386645; doi:10.1371/journal.pone.0236562)
Supplement: S2 Table — (DOCX) [file pone.0236562.s003.docx]

**Supplementary Table S2. Comparison between those in self-isolation versus not in self isolation**

|  | **Not in self-isolation** | | | **In self-isolation** | | |  |
| --- | --- | --- | --- | --- | --- | --- | --- |
|  | N | M | SD | N | M | SD | **Independent samples *t* test** |
| **Uncertain: future** | 2475 | 3.41 | 1.06 | 2592 | 3.52 | 1.08 | t (5065) = 3.63, p = 0.00 |
| **Lonely** | 2475 | 2.56 | 1.26 | 2592 | 2.76 | 1.29 | t (5065) = 5.52, p = 0.00 |
| **Worry: finances** | 2475 | 2.64 | 1.22 | 2592 | 2.78 | 1.27 | t (5065) = 4.09, p = 0.00 |
| **Worry: contracting COVID-19** | 2473 | 2.77 | 1.05 | 2580 | 2.91 | 1.08 | t (5051) = 4.65, p = 0.00 |
| **Perceived likelihood** | 2473 | 49.27 | 25.26 | 2584 | 47.27 | 24.40 | t (5055) = -2.86, p = 0.00 |
| **Perceived control** | 2473 | 70.16 | 20.36 | 2582 | 73.06 | 18.93 | t (5053) = 5.26, p = 0.00 |
| **Severity of illness** | 2467 | 3.18 | 0.94 | 2573 | 3.53 | 1.14 | t (5038) = 11.95, p = 0.00 |
| **Worry: loved ones contracting COVID-19** | 2475 | 3.44 | 1.04 | 2592 | 3.60 | 1.02 | t (5065) = 5.51, p = 0.00 |
| **Self-rated health** | 2339 | 3.10 | 0.94 | 2452 | 2.90 | 0.99 | t (4789) = 6.92, p = 0.00 |
| **DASS-21 Total** | 2461 | 38.05 | 24.44 | 2586 | 42.26 | 25.48 | t (5045) = 5.99, p = 0.00 |
| **DASS-21 Depression** | 2461 | 13.24 | 10.32 | 2586 | 15.01 | 10.72 | t (5045) = 5.97, p = 0.00 |
| **DASS-21 Anxiety** | 2461 | 8.15 | 7.85 | 2586 | 9.78 | 8.47 | t (5045) = 7.10, p = 0.00 |
| **DASS-21 Stress** | 2461 | 16.66 | 9.35 | 2586 | 17.47 | 9.60 | t (5045) = 3.03, p = 0.00 |
| **Whiteley-6 (health anxiety)** | 2470 | 12.27 | 5.20 | 2591 | 14.06 | 5.85 | t (5059) = 11.52, p = 0.00 |
| **Contamination Fears** | 2414 | 9.92 | 8.30 | 2514 | 11.56 | 9.14 | t (4926) = 6.60, p = 0.00 |
| **AUDIT-C Total (alcohol)** | 2358 | 3.25 | 2.63 | 2470 | 3.02 | 2.65 | t (4826) = -3.02, p = 0.00 |
| **PAVS Total (physical activity)** | 2362 | 190.10 | 296.41 | 2483 | 183.77 | 427.44 | t (4843) = -0.60, p = 0.55 |
