# Supplementary material for: Acute mental health responses during the COVID-19 pandemic in Australia
Source: PLoS One. 2020 Jul 28;15(7):e0236562. doi: 10.1371/journal.pone.0236562 (PMC7386645; doi:10.1371/journal.pone.0236562)
Supplement: Attachment — Submitted filename: Response to reviewers.docx [file pone.0236562.s005.docx]

**Reviewer #1: Overall, a well written and timely manuscript on an important topic. Main study limitations (e.g., limited sample representativeness) were identified and acknowledged. Findings of the exploratory analyses are of interest to a broad range of audiences. My main concern relates to the lack of information about the analytical procedures in the Methods section. Further comments and suggestions to strengthen the manuscript are as follows:**

**REVIEWER COMMENT: Introduction
- Further information about the COVID-19 containment regime and restrictions in place in Australia at the time (e.g., self-isolation, social distancing measures) could be provided (beyond the mandatory quarantine for overseas travelers) to better contextualise the study setting for readers.**

**RESPONSE:** We have included more information about the containment regimes, and restrictions put in place during the recruitment period on pages 5-6 of the revised manuscript.

**REVIEWER COMMENT:**

**Methods
- The Methods section does not appear to include any information on the types of ‘data analyses’ conducted (e.g., descriptive analyses, Independent samples t test, chi square analysis, linear regressions), which would be important to incorporate.**

**RESPONSE:** We thank the reviewer for pointing out this omission, and have included a detailed ‘Statistical analyses’ section in the revised manuscript. See page 10.

**Results
REVIEWER COMMENT: - (p.9, lines 213-14): suggest reordering to read: “The majority of participants rated their health as ‘good’ (37.7%), ‘very good’ (24.4%) or ‘fair’ (24.4%), …”**

**RESPONSE:** Thank you – we have edited this as suggested by the reviewer.

**REVIEWER COMMENT:**
**- (Table 1): decimal places for reported percentages could be kept consistent; for the Table to work standalone, it could include a footnote indicating the varied time-frames that mental health and general health measures referred to (e.g., over the past week, or lifetime)**

**RESPONSE:** We agree, and have changed the decimal places to one, to keep consistent in Table 1. We have extra information to clarify the time frames of the mental health diagnosis (lifetime), chronic illness (current), and self-rated health (general) in the Table 1 footnote.

**REVIEWER COMMENT:**

**- (Mental Health): The fact that more than 70% of respondents self-reported a lifetime mental health diagnosis strikes me as comparatively high and should be mentioned upfront in text when reporting sample descriptives. Likewise, 45% of respondents receiving current mental health treatment does seem equally elevated compared to general population stats and would also be worth reporting in text (as both have implications for sample representativeness).**

**RESPONSE:** We agree that there was a high proportion of respondents with previous history of mental health problems and were very surprised by this finding. We note that many previous self-report surveys of the acute mental health responses to pandemics (in the current crisis, and previous) have not asked about prior mental health diagnoses and could also be non-representative of the general population. As suggested, we have included more information in the sample description (pages 10-11). We also have a discussion of this issue in the Discussion section (pages 26-27; and in the limitations on page 30).

**REVIEWER COMMENT- (Table 4): row labels in the first column are difficult to read and could be reformatted**

**RESPONSE:** This has now been fixed, thank you for the feedback.

**REVIEWER COMMENT: - (p.20, line 299): please check finding – as ‘being unemployed’ does not seem to be significantly associated with depression (compare Table 8)**

**RESPONSE:** You are correct, thank you for noticing this error and drawing our attention to it. The error has now been fixed (see page 21).

**REVIEWER COMMENT:**

**Discussion
- (p.27, lines 443-448): while overarching AUDIT-C scores for this sample are indeed concerning, it could also be worth mentioning that those in self-isolation reported lesser alcohol consumption than those not self-isolating (compare Table 5)**

**RESPONSE:** This point has now been noted in the discussion (see page 29).

**REVIEWER COMMENT:** - **The striking finding that COVID variables accounted for very little variance in DASS scores would be worthy of further interpretation and discussion (e.g., in terms of potential methodological or conceptual implications)**

**RESPONSE:** We have included more discussion around this issue on page 28.

**REVIEWER COMMENT: Minor Typos
- (p.5, line 116): identify vulnerable groups who are [at] risk
- (p.7, line 153): hea[l]th
- (p.8, line 176): were administered in randomised [in] order to
- (p.26, line 416): was associate[d]**

**RESPONSE:** All of these typos have now been fixed, thank you.

**Reviewer #2: On the whole, this is a well-designed study of the psychological impact of the COVID-19 pandemic in Australia. The study authors have clearly made an attempt to extend the work of earlier authors, and to improve on it in some aspects.**

**REPSONSE**: Thank you.

**That being said, the following concerns still exist:

REVIEWER COMMENT:**

**1. Apart from the Chinese studies cited, a few studies from outside China have been published. It would be worth discussing these papers briefly in the Introduction or Discussion sections, to provide a broader context for the authors' work.**

**RESPONSE:** We agree, this is a rapidly evolving field, with new research being published quickly. We initially submitted our manuscript on the 1^st^ May 2020, and our literature review was up to date at the time of submission. Since then, others have been published, which we now briefly discuss in the Introduction and Discussion.

**REVIEWER COMMENT: 2. A measure of compulsive washing was included in the study. How did it correlate with measures of anxiety, depression or stress?**

**REPSONSE:** We conducted Pearson’s correlations between the compulsive washing subscale and measures of depression, anxiety and stress on the DASS-21. Correlations between the compulsive washing scale were r=.29 for DASS-21 depression, r=.40 for DASS-21 anxiety, and r=.36 for DASS-21 stress.

**REVIEWER COMMENT: 3. Given the small number of gender non-binary and Aboriginal / Islander subjects in the study, it may be premature to label these factors as robust predictors of mental health outcomes.**
 **REPSONSE:** We agree that the language should have been more tentative, given the small proportion of respondents who identified as non-binary gender identity or Aboriginal and/or Torres Strait Islander. Therefore we have amended the relevant section of the discussion (page 26) to specifically highlight the small number of these respondents, as follows:

*“There was a relatively small number of Indigenous Australians and respondents who identified as non-binary or different gender identity, or Indigenous Australians in the sample. However, this preliminary evidence suggests these groups may be vulnerable to poorer mental health during the current pandemic. Longitudinal research is needed to explore the longer term predictors of poorer mental health over time.”*

**REVIEWER COMMENT:**
**4. For the purpose of reading ease, it may be preferable to present only the key findings in tables and provide the remainder of the information as supplementary material.**

**RESPONSE:** Thank you for this suggestion. We have moved the tables describing the results of the comparisons between people with and without a pre-existing mental health diagnoses (previously Table 4) and the comparisons between those in self-isolation versus those who were not (previously Table 5) into the supplementary material – these are now S1 Table and S2 Table respectively.

**REVIEWER COMMENT:**
**Reviewer #3: The manuscript is well written. It addresses an important contemporary issue and provides information no available elsewhere in the literature. The key limitation in this manuscript is the sampling approach and the lack of a description and justification of the analysis plan.
* While the authors do address the sampling issues in their limitations briefly, I think that acknowledging this in more detail and presenting the findings in a more tentative manner would improve the publication.**

**RESPONSE:** We agree that although we believe it is very important to examine and report on the mental health outcomes of this cohort, given the sample characteristics the results may not be representative of the entire population. We have included more tentative language throughout the discussion, reporting on the high proportion of the people with lived experience of mental health problems in the results section to highlight this issue, and have included a more in-depth discussion of the issue of representativeness. We also have the planned analyses section in the methods, rather than the results.

**REVIEWER COMMENT: * The planned analyses are not described in the Methods. Additionally, inadequate justification is provided about why a step-wise approach to linear regression was adopted, and the rationale underlying the sequence and variables included at each step.**

**RESPONSE:** This has been fixed - see more information about the planned Statistical analyses section in the methods of the revised manuscript. See page 10.

**Methods > Recruitment

REVIEWER COMMENT: * Given that the sample that emerged does not appear representative of the Australian population generally it would be useful to include the survey advertisement as a supplementary material. The phrasing of the invitation to participate would foreseeably impact the sample composition.**

**RESPONSE:** In the revised manuscript, we have included the advertisements as supplementary material.

**REVIEWER COMMENT:**
*** Unless IP addresses external to Australia were blocked from survey participation it would be useful to describe in the results confirmation that all included IP addresses originated in Australia. Also, were any duplicate IP addresses identified? How were these managed?**

**RESPONSE:** The Qualtrics survey platform has an in-built tool/function that restricts multiple survey responses from the same IP address which enabled us to avoid duplicate IP entries. We did not check whether IP addresses originated in Australia. We have noted this in the limitations section of the revised manuscript (see page 30).

**REVIEWER COMMENT:**
Methods>Participants

*** Was any analysis completed comparing the completers versus the non-completers? It appears that any incomplete surveys were omitted from the data-set... how might this have biased the findings?**

**RESPONSE:** In the following analyses (and in the paper) we define completers as having 70% or more of the survey complete, whereas non-completers having less than 70% complete. In the non-completer group, we had too few participants with complete data on the DASS-21 (n=11) or the Whiteley health anxiety (n=20) to compare to the completer group. However, we did have sufficient numbers of participants to compare their scores on their age (n=279 non-completers), COVID-19 fears (n=204 non-completers) and risk perceptions (n-134 non-completers). There were no statistically significant differences between completers vs non completers on any of these variables, with one exception: participants who completed the survey had higher ratings of the extent to which their conversations had focused on COVID-19 in the past week (M= 3.52 vs M=3.37). While we cannot completely rule out bias because we do not have enough information on the non-completers (as most stopped shortly after the PISCF).

**REVIEWER COMMENT:**

*** Is the 15-minute completion timeframe based on actual participants or the estimation of the researchers? This is a relatively long survey and I am surprised by the completion rate. I wonder why this was so high - might it tell something about the sampling approach?**

**RESPONSE:** This was an estimate of the time taken to complete the survey. In addition we calculated the median estimated minutes to complete the survey and this was 15.9 minutes.

*** Did clicking on the link in social media open the PICF? Or did it open another page where potential participants needed to click to open the PICF? If the former it would be really helpful to include information about how many people clicked on the link without subsequently accessing the PICF.**

**REVIEWER COMMENT:**

**Methods > ****

Nil Analyses section is provided. It would be helpful to describe how the data was intended to be analysed a priori, and in particular the rationale for the step-wise approach to the regression.**

**RESPONSE:** We have included a new statistical analyses section in the revised manuscript (see page 10 of the revised manuscript) which also has the rationale for the stepwise regression approach.

**REVIEWER COMMENT**
**Results>Demographics

* The proportion of participants who identified as female gender and self-reported previous mental illness suggests that the sample is not representative. While this is discussed in the limitations I think it would be useful to consider this in greater detail in the Discussion. Why did the sampling approach result in such a high proportion of educated women with pre-existing mental illness participating? Similarly it suggests the relevance of more tentative language in the Discussion.**

**RESPONSE:** In the manscript, we had discussed the issue of representativeness in several points. We have included this information specifically in the sample demographics (see pages 10-11), and in the discussion (on page 25) and in the limitations (page 30).We have now included this additional sentence on p25 to specifically note the issue of representativeness of the general community: “…..*and that the sample was comprised of mostly women, and a high proportion of individuals with lived experience of mental health problems, and so is unlikely to be representative of the broader Australian general community.”* We have also added the term ‘associated with’ to apply more tentative language in the discussion.

**REVIEWER COMMENT:**
**Results>Health related information

* 4.8% of the sample reported having a friend of family member who had contracted COVID-19. Given the very low prevalence of this condition in Australia this again suggests an issue with the sampling that needs to be addressed in the limitations.**

**RESPONSE:** We have now included this in the limitation section of the revised manuscript, thank you for the suggestion (see p29).

**REVIEWER COMMENT: Results>Mental Health

* Symbol for statistical test at Line 251 is missing, presumably Chi squared**

**RESPONSE:** Thank you for pointing this out. It appears that when the word version was converted to the PDF version in the submission process, the chi squared symbol did not display correctly – it is however correct on the word version.

**REVIEWER COMMENT:**

**Results > Table 3

* Consider including the median for the DASS scores to given a clearer picture of central tendency**

**RESPONSE:** Thank you for this suggestion. We have included the median of all of the measures in Table 3 of the revised manuscript.

**REVIEWER COMMENT:**

**Results > Table 5

You have combined those in voluntary and required self-isolation. Were any analyses completed to see that the patter of results still held when the required self-isolation were excluded? Higher distress and disconnection would be foreseeable for this group.**

**RESPONSE**: As outlined above, as this analysis was not the primary aim of the study, on the recommendation of the reviewer we have included this analysis/table in the supplementary material. In hindsight, we would have provided a better definition of ‘forced’ versus ‘voluntary’ self-isolation. Because we did not provide a definition to participants in the questionnaire, and were unable to confirm whether people were in strictly enforced self isolation (from the NSW Health, or the relevant authorities) we therefore chose to include all of those who reported being in isolation in the same group. We believe would be an important area for future research in countries where there are larger numbers of participants required to self-isolate.  **REVIEWER COMMENT:**

**Discussion:

See general comments about the need to discuss sampling issues in more detail and present findings more tentatively.**

**RESPONSE:** We have amended the revised discussion to use more tentative language given lack of representativeness of the sample, thank you.
